# Supplementary material for: Allosteric regulation and crystallographic fragment screening of SARS-CoV-2 NSP15 endoribonuclease
Source: Nucleic Acids Res. 2023 Apr 28;51(10):5255–70. doi: 10.1093/nar/gkad314 (PMC10250223; doi:10.1093/nar/gkad314)
Supplement: gkad314_Supplemental_Files [file gkad314_supplemental_files.zip › Supplementary datasets.zip!/160/Refine_8/01-BUSTER/Cycle-2/shell.01/R_Rfree.html]

# Rxpct factors in resolution bins at cycle 0


|  |  |  |  |  |  |  |  |  |
| --- | --- | --- | --- | --- | --- | --- | --- | --- |
|  | **<d> (Å)**  Rxpct\_Work | Rxpct\_Free | Rxpct\_All | |  |  |  |  |
|  | 14.6046 | 0.3892 | 0.5910 | 0.3963 |  |
|  | 8.4320 | 0.2456 | 0.2741 | 0.2472 |  |
|  | 6.5314 | 0.2131 | 0.2625 | 0.2158 |  |
|  | 5.5200 | 0.1829 | 0.2321 | 0.1849 |  |
|  | 4.8682 | 0.1494 | 0.1948 | 0.1519 |  |
|  | 4.4035 | 0.1524 | 0.1839 | 0.1542 |  |
|  | 4.0506 | 0.1590 | 0.1847 | 0.1602 |  |
|  | 3.7709 | 0.1676 | 0.2514 | 0.1721 |  |
|  | 3.5421 | 0.1725 | 0.2117 | 0.1745 |  |
|  | 3.3505 | 0.1771 | 0.2479 | 0.1809 |  |
|  | 3.1870 | 0.1813 | 0.2396 | 0.1845 |  |
|  | 3.0453 | 0.1839 | 0.2270 | 0.1858 |  |
|  | 2.9209 | 0.1922 | 0.2057 | 0.1928 |  |
|  | 2.8107 | 0.1878 | 0.2387 | 0.1902 |  |
|  | 2.7120 | 0.1904 | 0.2206 | 0.1920 |  |
|  | 2.6231 | 0.1988 | 0.2145 | 0.1997 |  |
|  | 2.5423 | 0.2088 | 0.2232 | 0.2096 |  |
|  | 2.4686 | 0.2115 | 0.2225 | 0.2121 |  |
|  | 2.4010 | 0.2225 | 0.2293 | 0.2228 |  |
|  | 2.3386 | 0.2505 | 0.2600 | 0.2509 |  |
|  | **Overall** | 0.1911 | 0.2347 | 0.1933 |  |

# Rxpct factors in resolution bins at cycle 1


|  |  |  |  |  |  |  |  |  |
| --- | --- | --- | --- | --- | --- | --- | --- | --- |
|  | **<d> (Å)**  Rxpct\_Work | Rxpct\_Free | Rxpct\_All | |  |  |  |  |
|  | 14.6046 | 0.3895 | 0.5915 | 0.3966 |  |
|  | 8.4320 | 0.2458 | 0.2741 | 0.2475 |  |
|  | 6.5314 | 0.2133 | 0.2627 | 0.2160 |  |
|  | 5.5200 | 0.1830 | 0.2321 | 0.1850 |  |
|  | 4.8682 | 0.1494 | 0.1950 | 0.1519 |  |
|  | 4.4035 | 0.1525 | 0.1840 | 0.1542 |  |
|  | 4.0506 | 0.1590 | 0.1847 | 0.1602 |  |
|  | 3.7709 | 0.1673 | 0.2512 | 0.1718 |  |
|  | 3.5421 | 0.1723 | 0.2116 | 0.1743 |  |
|  | 3.3505 | 0.1769 | 0.2478 | 0.1808 |  |
|  | 3.1870 | 0.1812 | 0.2393 | 0.1844 |  |
|  | 3.0453 | 0.1838 | 0.2269 | 0.1857 |  |
|  | 2.9209 | 0.1922 | 0.2057 | 0.1928 |  |
|  | 2.8107 | 0.1878 | 0.2392 | 0.1902 |  |
|  | 2.7120 | 0.1903 | 0.2205 | 0.1919 |  |
|  | 2.6231 | 0.1986 | 0.2145 | 0.1995 |  |
|  | 2.5423 | 0.2087 | 0.2226 | 0.2094 |  |
|  | 2.4686 | 0.2114 | 0.2223 | 0.2120 |  |
|  | 2.4010 | 0.2225 | 0.2292 | 0.2228 |  |
|  | 2.3386 | 0.2505 | 0.2603 | 0.2510 |  |
|  | **Overall** | 0.1910 | 0.2347 | 0.1932 |  |

# Rxpct factors in resolution bins at cycle 2


|  |  |  |  |  |  |  |  |  |
| --- | --- | --- | --- | --- | --- | --- | --- | --- |
|  | **<d> (Å)**  Rxpct\_Work | Rxpct\_Free | Rxpct\_All | |  |  |  |  |
|  | 14.6046 | 0.3898 | 0.5920 | 0.3969 |  |
|  | 8.4320 | 0.2461 | 0.2742 | 0.2477 |  |
|  | 6.5314 | 0.2135 | 0.2629 | 0.2162 |  |
|  | 5.5200 | 0.1831 | 0.2321 | 0.1851 |  |
|  | 4.8682 | 0.1494 | 0.1952 | 0.1520 |  |
|  | 4.4035 | 0.1525 | 0.1841 | 0.1542 |  |
|  | 4.0506 | 0.1589 | 0.1848 | 0.1601 |  |
|  | 3.7709 | 0.1670 | 0.2510 | 0.1715 |  |
|  | 3.5421 | 0.1721 | 0.2115 | 0.1741 |  |
|  | 3.3505 | 0.1769 | 0.2477 | 0.1807 |  |
|  | 3.1870 | 0.1811 | 0.2389 | 0.1843 |  |
|  | 3.0453 | 0.1837 | 0.2270 | 0.1856 |  |
|  | 2.9209 | 0.1922 | 0.2059 | 0.1928 |  |
|  | 2.8107 | 0.1877 | 0.2397 | 0.1901 |  |
|  | 2.7120 | 0.1903 | 0.2203 | 0.1919 |  |
|  | 2.6231 | 0.1984 | 0.2148 | 0.1994 |  |
|  | 2.5423 | 0.2086 | 0.2223 | 0.2094 |  |
|  | 2.4686 | 0.2113 | 0.2222 | 0.2119 |  |
|  | 2.4010 | 0.2224 | 0.2293 | 0.2227 |  |
|  | 2.3386 | 0.2506 | 0.2602 | 0.2510 |  |
|  | **Overall** | 0.1910 | 0.2347 | 0.1932 |  |

# Rxpct factors in resolution bins at cycle 3


|  |  |  |  |  |  |  |  |  |
| --- | --- | --- | --- | --- | --- | --- | --- | --- |
|  | **<d> (Å)**  Rxpct\_Work | Rxpct\_Free | Rxpct\_All | |  |  |  |  |
|  | 14.6046 | 0.3855 | 0.5852 | 0.3925 |  |
|  | 8.4320 | 0.2470 | 0.2751 | 0.2487 |  |
|  | 6.5314 | 0.2173 | 0.2607 | 0.2197 |  |
|  | 5.5200 | 0.1853 | 0.2430 | 0.1877 |  |
|  | 4.8682 | 0.1502 | 0.2047 | 0.1533 |  |
|  | 4.4035 | 0.1517 | 0.1866 | 0.1537 |  |
|  | 4.0506 | 0.1556 | 0.1854 | 0.1569 |  |
|  | 3.7709 | 0.1619 | 0.2396 | 0.1661 |  |
|  | 3.5421 | 0.1670 | 0.2014 | 0.1687 |  |
|  | 3.3505 | 0.1745 | 0.2434 | 0.1782 |  |
|  | 3.1870 | 0.1795 | 0.2361 | 0.1827 |  |
|  | 3.0453 | 0.1820 | 0.2308 | 0.1842 |  |
|  | 2.9209 | 0.1925 | 0.2135 | 0.1934 |  |
|  | 2.8107 | 0.1875 | 0.2444 | 0.1901 |  |
|  | 2.7120 | 0.1913 | 0.2189 | 0.1927 |  |
|  | 2.6231 | 0.1984 | 0.2175 | 0.1996 |  |
|  | 2.5423 | 0.2102 | 0.2195 | 0.2107 |  |
|  | 2.4686 | 0.2105 | 0.2245 | 0.2112 |  |
|  | 2.4010 | 0.2221 | 0.2328 | 0.2226 |  |
|  | 2.3386 | 0.2518 | 0.2694 | 0.2526 |  |
|  | **Overall** | 0.1899 | 0.2347 | 0.1922 |  |
